# Supplementary figures and images for: Genetic Diversity and Population Structure Analysis of Castanopsis hystrix and Construction of a Core Collection Using Phenotypic Traits and Molecular Markers
Source: Genes (Basel). 2022 Dec 16;13(12):2383. doi: 10.3390/genes13122383 (PMC9778198; doi:10.3390/genes13122383)

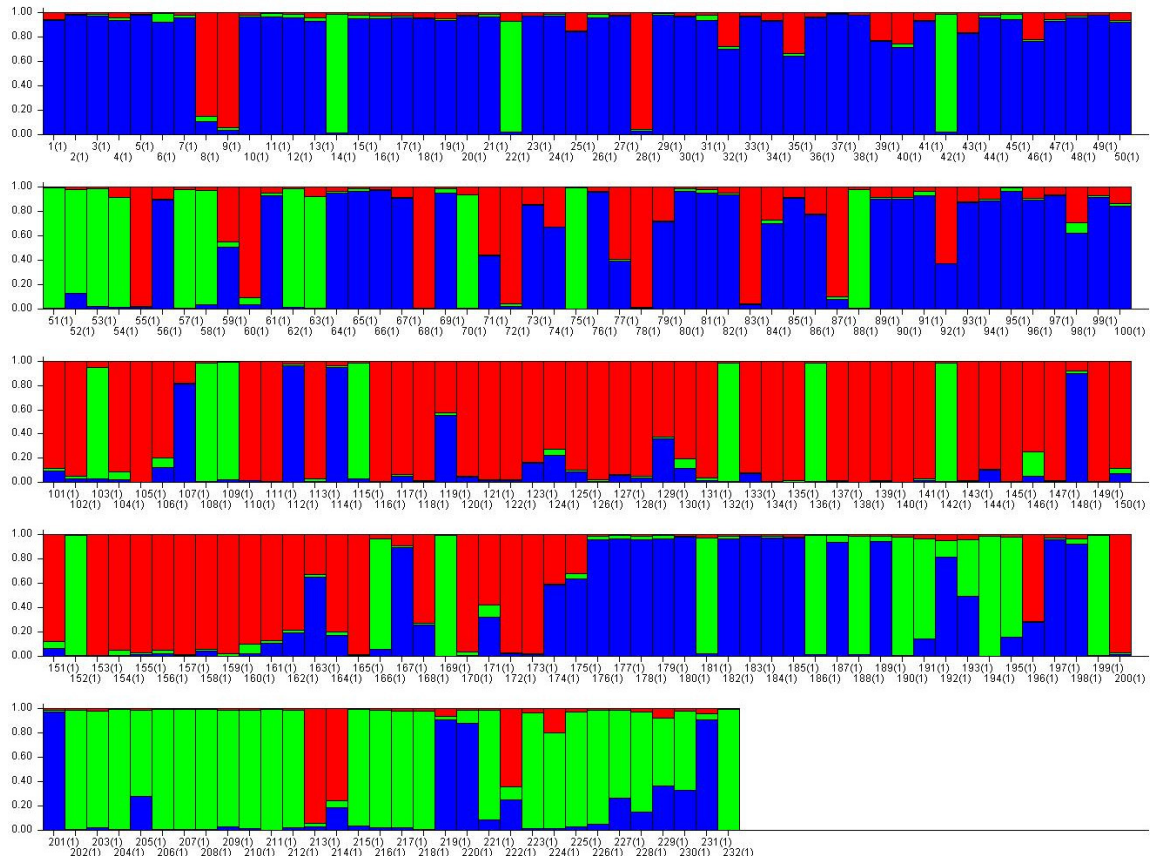

**Figure S1:** The classification results of structure by the STRUCTURE

Supplement: Supplementary file 1 [file genes-13-02383-s001.zip › Supplementary-Figure S1.pdf]
